# Study of the Expression of Pain and Inflammation Pathway Genes and Their Associated miRNAs in Patients With Endometriosis

**DOI:** 10.1155/bmri/9911726

**Published:** 2025-10-13

**Authors:** Azam Govahi, Samaneh Rokhgireh, Fateme Arjmand, Mahmood Barati, Marziyeh Ajdary, Shahla Chaichian, Shahla Mirgaloybayat

**Affiliations:** ^1^ Endometriosis Research Center, Iran University of Medical Sciences (IUMS), Tehran, Iran, iums.ac.ir; ^2^ Reproductive Sciences and Technology Research Center, Iran University of Medical Sciences, Tehran, Iran, iums.ac.ir; ^3^ Department of Biotechnology, Faculty of Allied Medicine, Iran University of Medical Sciences (IUMS), Tehran, Iran, iums.ac.ir; ^4^ Iranian Scientific Society of Minimally Invasive Gynecology, Tehran, Iran; ^5^ Department of Obstetrics and Gynecology, School of Medicine, Iran University of Medical Sciences, Tehran, Iran, iums.ac.ir

**Keywords:** *CGRP*, *ENA-78*, endometriosis, miRNA

## Abstract

**Introduction:**

Inflammatory mechanisms mediate neuropathies in patients with endometriosis. The *ENA-78* and *CGRP* genes are involved in pain signaling and inflammatory responses in these patients. However, the regulatory role of miRNAs targeting these genes remains unknown. This study is aimed at investigating the expression of *CGRP*‐ and *ENA-78*‐related miRNAs in plasma and endometrial tissues in women with endometriosis and comparing it with healthy fertile women.

**Materials and Methods:**

Blood plasma, ectopic (EC), and eutopic (EU) endometrium samples from 30 patients with endometriosis and blood plasma and endometrial tissue samples from 30 healthy fertile women were collected. The Q‐PCR technique was used to determine the expression levels of *ENA-78* and *CGRP* genes and their associated microRNAs.

**Results:**

Expression of the *CGRP* gene in EC and EU tissues of the endometriosis group increased compared to the control group, and the expression of miRNAs related to this gene (*hsa-miR-5584-5p* and *hsa-miR-410-5p*) in EC and EU tissues and serum of endometriosis patients decreased compared to the control group. Also, the expression of the *ENA-78* gene in EC and EU tissues of the endometriosis group increased compared to the control group, and the expression of miRNAs related to this gene (*hsa-miR-4748* and *hsa-miR-92a-3p*) in EC and EU tissues and serum of endometriosis patients decreased compared to the control group (*p* < 0.05).

**Conclusion:**

Identification of miRNAs related to the pain and inflammation pathway may provide us with new markers for the evaluation of endometriosis. *hsa-miR-5584-5p*, *hsa-miR-410-5p*, *hsa-miR-4748*, and *hsa-miR-92a-3p* modulate neuroimmune responses in endometriosis and may serve as future diagnostic or therapeutic targets.

## 1. Introduction

Endometriosis affects approximately 10% of all women of reproductive age, and its prevalence increases to 20%–50% in infertile women [1]. To standardize the assessment of disease severity and facilitate treatment decisions, various systems have been developed, one of the most widely used of which is the American Society for Reproductive Medicine (ASRM) classification system. Introduced in 1996, this system divides endometriosis into four stages (I to IV) based on clinical and surgical criteria. The main scoring criteria include the extent and depth of endometriotic implants, the presence and severity of pelvic adhesions, and ovarian involvement [2].

In this disease, endometrial cells invade and proliferate outside the uterus, forming endometriotic implants. These implants act like the normal (eutopic) endometrium; that is, they proliferate with the hormonal cycle and undergo periodic bleeding. The activity of these implants leads to painful internal scarring, dysfunction of the related organ, and the formation of intratissue adhesions [3]. Endometriosis can occur for various reasons, such as hormonal [4], immunological [5], environmental and toxic pollutants [6, 7], genetic [7], and, finally, epigenetic [7]. In some cases, endometriosis is accompanied by severe pain attacks. Among the neurotransmitters involved in pain is calcitonin gene–related peptide (*CGRP*), which plays a role in the development of acute pain episodes [8]. Since endometriosis pain is often in the form of cyclic dysmenorrhea, it seems that hormonal fluctuations during the monthly cycle contribute to the development of attacks. So far, studies have shown that CGRP levels are related to estrogen levels. However, *CGRP* levels in women have not yet been determined during the menstrual cycle. Along with the proliferation and growth of endometriotic cells, *CGRP* seems to cause neurogenic inflammation and reduce NO levels, contributing to the development or increase of pain [9–11]. Another important marker that is effective in the progression of pain is epithelial‐derived neutrophil‐activating peptide 78 (*ENA-78*), which activates cytokines and chemokines and promotes inflammation, which subsequently increases the patient’s pain perception [12]. Studies have shown that *ENA-78* activates *CXCR2* and promotes the progression of endometriosis and inflammation [13, 14].

MicroRNAs are noncoding RNAs that are typically 18–25 nucleotides long and regulate gene expression by degrading or blocking the translation of target mRNA. These molecules exert their action by base‐pairing to the 3 ^′^ untranslated region (3 ^′^UTR) of the target mRNA [15]. MicroRNAs play important roles in numerous pathological diseases and physiological disorders. Each microRNA controls the translation of many genes simultaneously. However, despite the fact that microRNAs are involved in the pathogenesis of endometriosis by regulating multiple transcripts related to hypoxia, inflammation, apoptosis, tissue repair, cell proliferation, extracellular matrix remodeling, and angiogenesis, information on circulating microRNAs in blood and endometriosis is limited [15–17]. Some microRNAs target genes involved in implantation and can prevent successful implantation [18].

Overall, our hypothesis in this study is that in endometriosis, the combination of hypoxia, oxidative stress (caused by iron accumulation), and chronic inflammation creates a vicious cycle that increases the expression of *ENA-78* and *CGRP* through the activation of the transcription factors *HIF-1α* and *NF-κB*. *ENA-78* promotes tissue destruction and further cytokine release by attracting neutrophils [8, 19]. At the same time, *CGRP*, by stimulating neuroinflammation, vasodilation, and nerve growth, both exacerbates the inflammatory environment and directly contributes to the generation of pain. This two‐way (inflammation–nerve) interaction facilitates the survival and progression of endometriotic lesions.

Therefore, the aim of this study was to investigate the expression of microRNAs related to the *CGRP* and *ENA-78* genes in EU and EC tissues and serum of individuals with endometriosis in order to identify changes in these microRNAs and use them in diagnosis and treatment.

## 2. Materials and Methods

### 2.1. Ethics Statement

The Institutional Ethics Committee of Iran University of Medical Sciences approved this study (IR.IUMS.REC.1402.994), and after obtaining informed consent from the patients, tissue samples were collected from the tissue scraps sent to the pathology laboratory. The samples were included in the study without mentioning the patient’s name, and the Helsinki principle of confidentiality was observed.

### 2.2. Tissue Collection

Thirty women with endometriosis who were candidates for diagnostic laparoscopy were selected from Rasoul Akram Hospital, Iran University of Medical Sciences; the indication for endometriosis surgery included bowel, ureter, bladder, and ovarian involvement, which caused dyspepsia, hydronephrosis, hematuria, and pain, respectively. If the patient had ovarian cysts, egg freezing was performed before endometriosis surgery at the request of the infertility clinic. All participating patients had Endometriosis Grade III, IV according to the ASRM classification system [2]. Thirty fertile women who had undergone diagnostic laparoscopy and had no signs of pain and endometriosis in their pelvis were selected as the control group. All patients were in the age range of 20–45 years and did not receive any hormones for 3 months before surgery. Peripheral blood samples were also taken from all patients in the proliferative phase of the menstrual cycle in the operating room before surgery. Tissue samples were confirmed by a pathologist according to the criteria of Noyes et al. [20]; there was no specific criterion for confirming EC and EU samples. The pathologist considered samples with a large number of pigmented histiocytes and endometrial stroma and a small number of glands as inactive endometrial samples, which were excluded from the study [21]. Endometrial tissue samples from the control group and EC and EU tissues from the endometriosis group were collected and stored at −80°C to examine the expression of *ENA-78*, *CGRP*, and related miRNAs.

### 2.3. Blood Sample Processing

Peripheral blood of all patients was centrifuged for 10 min at 3000 g in sterile tubes containing coagulant, and plasma was separated; then, plasma was stored at −80°C to examine the expression of miRNAs related to the *CGRP* and *ENA-78* genes.

### 2.4. Selection of miRNAs Related to the CGRP and ENA‐78 Genes

The miRWalk, miRPara, and TargetScan databases were used to select miRNAs related to the genes. Each miRNA with the highest score for binding to mRNA was selected. For the *CGRP* gene, the miRNAs *hsa-miR-5584-5p* and *hsa-miR-410-5p* were selected, and for the *ENA-78* gene, the miRNAs *hsa-miR-4748* and *hsa-miR-92a-3p* were selected.

### 2.5. Real‐Time Reverse Transcription–Polymerase Chain Reaction (RT‐PCR)

To perform the Q‐PCR technique, RNA was first extracted from whole tissues and plasma using TRIzol TRI (Sigma‐Aldrich), and cDNA synthesis was performed using a kit (Thermo Fisher Scientific Company, Japan, MAN0012612, K1612); the primers used in the study are listed in Table 1. Primer design was done using the stem loop method, and their Tm and interaction were examined with NCBI primer blast. The cDNA samples, primers used for genes and miRNAs, were used for real‐time RT‐PCR, and all melting curves were dimer‐free. For cDNA synthesis, 1 *μ*g of RNA was used per reaction. In the RT‐PCR step, 1 *μ*L of cDNA (approximately equivalent to 20–50 ng cDNA) was used per reaction. To perform Q‐PCR, 1 *μ*L cDNA, 1 *μ*L of each primer (20 pmol), 5.5 *μ*L molecular weight water, and 2.5 *μ*L SYBR Green were used.

**Table 1:** Primers used in this study.

**Name**	**Sequence (5** ^′^ **⟶3** ^′^ **)**
*hsa-miR-410-5p* -RT-Chen	5 ^′^ -GTCGTATCCAGTGCAGGGTCCGAGGTATTCGCACTGGATACGACCGAACT-3 ^′^
*hsa-miR-410-5p* -F-Chen	5 ^′^ -CGTCGAGGTTGTCTGTGATG-3 ^′^
RG-Chen	5 ^′^ -CAGTGCAGGGTCCGAGGTA-3 ^′^
*hsa-miR-5584-5p* -RT-Chen	5 ^′^ -GTCGTATCCAGTGCAGGGTCCGAGGTATTCGCACTGGATACGACTCTAGT-3 ^′^
*hsa-miR-5584-5p* -F-Chen	5 ^′^ -AAGCGCAGGGAAATGGGAAG-3 ^′^
*hsa-miR-4748* -RT-Chen	5 ^′^ -GTCGTATCCAGTGCAGGGTCCGAGGTATTCGCACTGGATACGACAGCAAA-3 ^′^
*sa-miR-4748* -F-Chen	5 ^′^ -GTGGGAGGTTTGGGGAGG-3 ^′^
*hsa-miR-92a-3p*‐RT‐Chen	5 ^′^ -GTCGTATCCAGTGCAGGGTCCGAGGTATTCGCACTGGATACGACACAGGC-3 ^′^
*hsa-miR-92a-3p* -F-Chen	5 ^′^ -AAGCGTATTGCACTTGTCCC-3 ^′^
CGRP (*CALCA*) forward	5 ^′^‐CCATGCAGCACCATTCAGGTC‐3 ^′^
CGRP (*CALCA*) reverse	5 ^′^‐TGGAGCCCTCTCTCTCTTGC‐3 ^′^
ENA‐78 (*CXCL5*) forward	5 ^′^‐AGAGAGCTGCGTTGCGTTTG‐3 ^′^
ENA‐78 (*CXCL5*) reverse	5 ^′^‐TCTTCAGGGAGGCTACCACTTC‐3 ^′^
U6‐RT	5 ^′^‐GTCGTACTCAACGTGGTTAGGGTCCGAGGTATAGGTT‐3 ^′^
	5 ^′^‐CCCACGTGGAGGACGACGAATATG‐3 ^′^
U6‐F	5 ^′^‐GGATGACGCAAATTCGTGAAGC‐3 ^′^
*Actin, beta (.)-F*	5 ^′^‐CAAGATCATTGCTCCTCCTG‐3 ^′^
*Actin, beta (β-actin)-R*	5 ^′^‐ATCCACATCTGCTGGAAGG‐3 ^′^

Each reaction contained the following:

• 1 *μ*L cDNA (equivalent to ~20–50 ng of total RNA input)
• 1 *μ*L of each forward and reverse primer (20 pmol/*μ*L) (Table 1)
• 5.5 *μ*L nuclease‐free water
• 2.5 *μ*L SYBR Green PCR Master Mix
• Total reaction volume: 10 *μ*L

The thermal cycling conditions were as follows:

• Initial denaturation: 95°C for 5 min
• Amplification (40 cycles):
  o. Denaturation: 95°C for 30 s
  o. Annealing: primer annealing temperature, 60°C for 30 s
  o. Extension: 72°C for 30 s
• Melting curve analysis: 55°C to 95°C (increment: 0.5°C per step)

The difference in cycle time (*Δ*CT) between the expression levels of genes, miRNA, and Housekeeping was defined. miRNAs and genes were normalized by U6 and *β*‐actin internal controls, respectively. The method was performed as a difference between patients and controls using the 2^−*Δ* *Δ*CT^ method [22].

### 2.6. Statistical Analysis

Statistical data of each group were analyzed using the ANOVA test and SPSS software Version 24.0 (IBM). For comparison between groups, a post hoc *t*‐test was used. *p* < 0.05 was considered significant. All data were presented as mean ± SD.

## 3. Results

### 3.1. CGRP Gene Expression in Tissue

**Figure 1:**
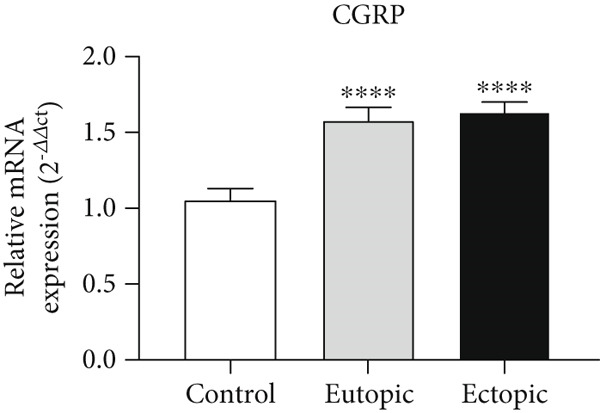
Gene expression array analysis for the *CGRP* gene. There are three biological replicates in each group. mRNA levels were examined using qRT‐PCR. Data are presented as mean ± SD (*n* = 30 per group). Internal control was provided with *β*‐actin.  ^∗∗∗^ *p* < 0.0001.

As shown in Figure 1, *CGRP* gene expression was increased in the EU (1.56 ± 0.08) and EC groups (1.61 ± 0.07) compared to the control group (1.05 ± 0.06) (*p* < 0.0001).

### 3.2. ENA‐78 Gene Expression in Tissue

**Figure 2:**
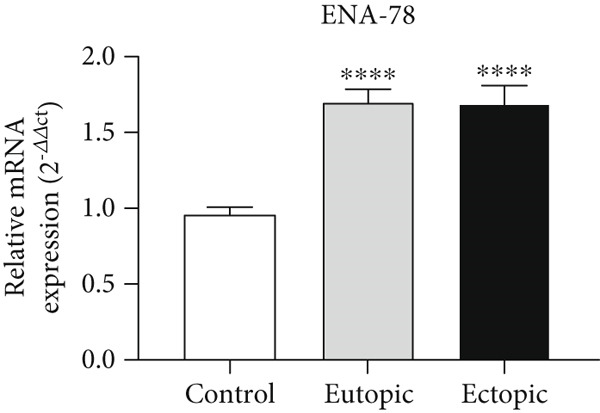
Gene expression array analysis for the *ENA-78* gene. There are three biological replicates in each group. mRNA levels were examined using qRT‐PCR. Data are presented as mean ± SD (*n* = 30 per group). Internal control was provided with *β*‐actin.  ^∗∗∗^ *p* < 0.0001.

As shown in Figure 2, *ENA-78* gene expression was increased in the EU (1.70 ± 0.08) and EC groups (1.69 ± 0.11) compared to the control group (0.96 ± 0.05) (*p* < 0.0001).

### 3.3. Expression Levels of MicroRNAs hsa‐miR‐5584‐5p and hsa‐miR‐410‐5p Related to the CGRP Gene at the Tissue Level

According to Figure 3, the expression of *hsa-miR-5584-5p* was decreased in EC (0.92 ± 0.24) and EU (0.89 ± 0.25) tissue of the endometriosis group compared to the control group (1.06 ± 0.11) (*p* < 0.05). The expression of *hsa-miR-410-5p* was decreased in EC (0.89 ± 0.22) and EU (0.89 ± 0.27) tissue of the endometriosis group compared to the control group (1.08 ± 0.09), but it was not statistically significant.

Figure 3MicroRNA expression array analysis of (a) *hsa-miR-5584-5p* and (b) *hsa-miR-410-5p* at the tissue level. There were three biological replicates in each group. mRNA levels were examined using qRT‐PCR. Data are presented as mean ± SD (*n* = 30 per group). Internal control was provided with *β*‐actin.  ^∗^ *p* < 0.05.

**Figure:**
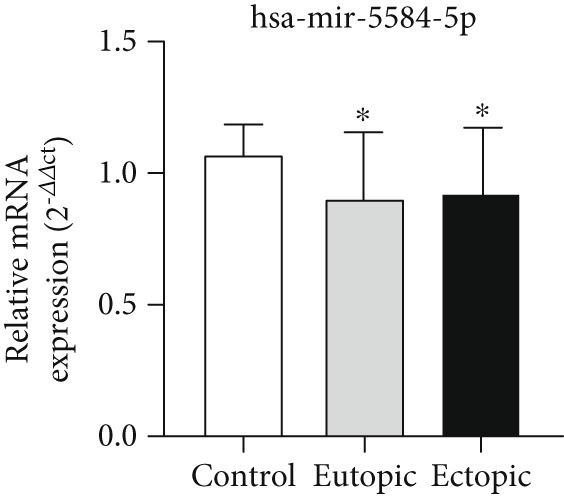
(a)

**Figure:**
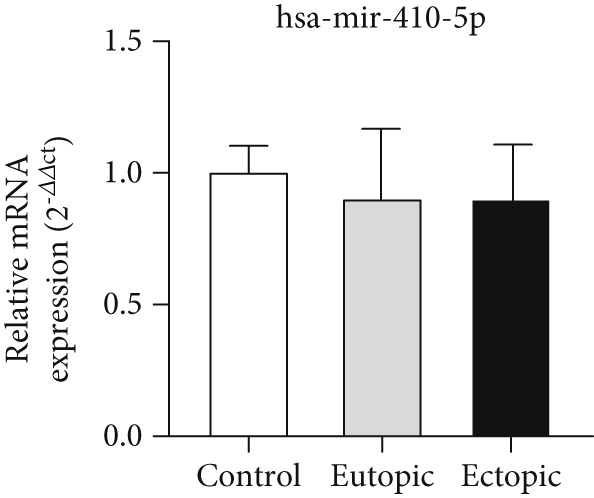
(b)

### 3.4. Expression Levels of MicroRNAs hsa‐miR‐5584‐5p and hsa‐miR‐410‐5p Related to the CGRP Gene at the Serum Level

According to Figure 4, the expression of *hsa-miR-5584-5p* in the serum of the endometriosis group (0.99 ± 0.30) decreased compared to the control group (1.01 ± 0.09), but it was not statistically significant. According to Figure 4, the expression of *hsa-miR-410-5p* in the serum of the endometriosis group (0.97 ± 0.39) decreased compared to the control group (1.11 ± 0.06), but it was not statistically significant.

Figure 4MicroRNA expression array analysis of (a) *hsa-miR-5584-5p* and (b) *hsa-miR-410-5p* at the serum level. There are three biological replicates in each group. mRNA levels were examined using qRT‐PCR. Data are presented as mean ± SD (*n* = 30 per group). Internal control was provided with *β*‐actin.

**Figure:**
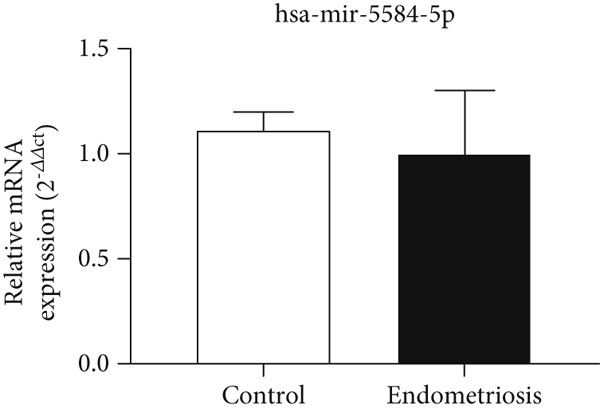
(a)

**Figure:**
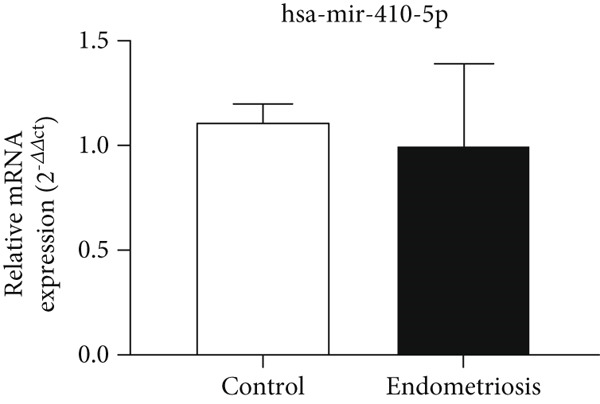
(b)

### 3.5. Expression Levels of MicroRNAs hsa‐miR‐4748 and hsa‐miR‐92a‐3p Related to the ENA‐78 Gene at the Tissue Level

According to Figure 5, the expression of *hsa-miR-4748* was decreased in the EC (0.87 ± 0.10) and EU (0.87 ± 0.10) tissue levels of the endometriosis group compared to the control group (0.94 ± 0.09) (*p* < 0.05).

Figure 5MicroRNA expression array analysis of (a) *hsa-miR-4748* and (b) *hsa-miR-92a-3p* at the tissue level. There are three biological replicates in each group. mRNA levels were examined using qRT‐PCR. Data are presented as mean ± SD (*n* = 30 per group). Internal control was provided with *β*‐actin.  ^∗^ *p* < 0.05.

**Figure:**
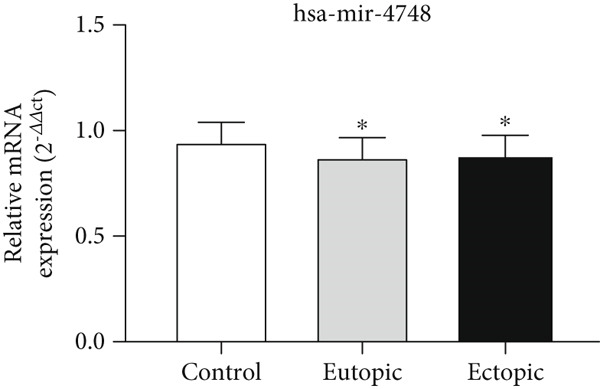
(a)

**Figure:**
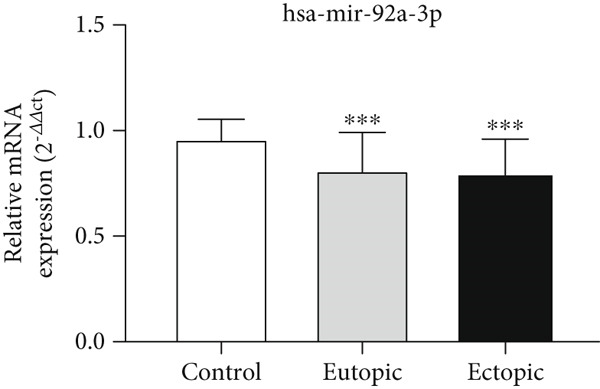
(b)

According to Figure 5, the expression of *hsa-miR-92a-3p* was decreased in the EC (0.79 ± 0.17) and EU (0.80 ± 0.18) tissue levels of the endometriosis group compared to the control group (0.96 ± 0.08) (*p* < 0.05).

### 3.6. Expression Levels of MicroRNAs hsa‐miR‐4748 and hsa‐miR‐92a‐3p Related to the ENA‐78 Gene at the Serum Level

According to Figure 6, the expression of *hsa-miR-4748* in the serum of the endometriosis group (0.85 ± 0.26) was decreased compared to the control group (0.95 ± 0.06) (*p* < 0.05). According to Figure 6, the expression of *hsa-miR-92a-3p* in the serum of the endometriosis group (0.84 ± 0.16) was decreased compared to the control group (0.91 ± 0.09) (*p* < 0.05).

Figure 6MicroRNA expression array analysis of (a) *hsa-miR-4748* and (b) *hsa-miR-92a-3p* at the serum level. There are three biological replicates in each group. mRNA levels were examined using qRT‐PCR. Data are presented as mean ± SD (*n* = 30 per group). Internal control was provided with *β*‐actin.  ^∗^ *p* < 0.05.

**Figure:**
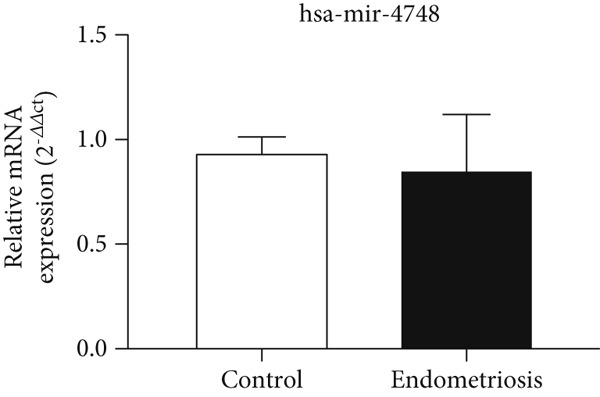
(a)

**Figure:**
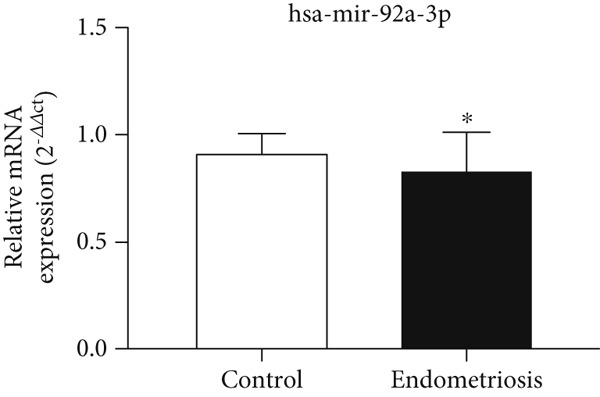
(b)

## 4. Discussion

The results of this study showed that the expression of the *CGRP* gene in EC and EU tissues of the endometriosis group increased compared to the control group, and the expression of miRs related to this gene (hsa‐miR‐5584‐5p) in EC and EU tissues of endometriosis patients decreased compared to the control group. Also, the expression of the ENA‐78 gene in EC and EU tissues of the endometriosis group increased compared to the control group, and the expression of miRNAs related to this gene (*hsa-miR-4748* and *hsa-miR-92a-3p*) in EC and EU tissues and serum of endometriosis patients decreased compared to the control group.

*CGRP* expression is associated with estrogen levels. In fact, high estrogen increases *CGRP* transcription by suppressing MAPK and *TNF-α* signaling pathways, and both *CGRP* expression increases and apoptosis rates decrease [8, 23].

In the present study, *CGRP* expression was significantly increased in endometriosis patients, which, given the high estrogen levels in these patients, could indicate a relationship between *CGRP* expression and estrogen levels.

A study showed that *miR-410-5p* induces apoptosis by targeting PIM1, inhibits obesity‐associated cardiac remodeling in patients with heart failure by suppressing Smad7 and activating TGF‐*β*/Smad, and inhibits mitophagy in ischemia/reperfusion injury by downregulating HMGB1 [24]. This study demonstrated that *miR-410-5p* is effective in modulating cell fate, inflammation, and tissue regeneration. However, *miR-5584-5p* remains largely unknown; our data suggest it as a promising candidate in endometriosis research.

The results of this study showed that *ENA-78* was overexpressed in EC and EU tissues. Consistent with these results, it has been reported that *ENA-78* levels are increased in peritoneal fluid and plasma of women with pelvic pain [19]. *ENA-78* is a chemokine for neutrophil recruitment and cytokine activation [19] and is effective in local inflammation and pain signaling [13]. It has been reported that high levels of *ENA-78*, together with proinflammatory cytokines TNF‐*α* and IL‐6, in the follicular fluid of infertile patients with endometriosis disrupt the follicular microenvironment [25]. In the present study, a significant decrease in the regulatory miRNAs *miR-92a-3p* and *miR-4748* was observed in patients with endometriosis, which is consistent with the increased expression of *ENA-78* in these patients. Zhu et al. reported that high expression of miR‐92a‐3p can inhibit proliferation and invasion and suppress the progression of endometriosis through the modulation of the TCF21/SF‐1 signaling axis [26]. In addition, Li et al. reported that miR‐92a‐3p is involved in progesterone resistance by targeting PTEN, and its inhibition enhances the therapeutic efficacy of progesterone [27]. The dual role of miR‐92a‐3p in both suppressing cell invasion and enhancing hormonal resistance highlights its effect in controlling endometriosis. The results of this study are also consistent with another study showing reduced levels of *miR-92a-3p* in the circulation and tissue in patients with endometriosis [28], highlighting its importance as a potential diagnostic biomarker. Importantly, reduced *miR-92a-3p* in endometriosis patients leads to increased levels of *ENA-78*, increased recruitment of neutrophils and leukocytes, and exacerbated inflammatory conditions in these patients.

The results of this study showed a decrease in the expression of *miR-4748* in endometriosis patients. Although miR‐4748 is less well known, considering its effect on the reduction of *ENA-78* expression, its reduction in the tissue and serum of endometriosis patients could lead to an increase in *ENA-78* expression and promote inflammatory conditions. Also, its reduction in expression in endometriosis patients may play a role in the dysregulation of local immune or hormonal pathways. Given the predicted role of microRNAs in regulating the cell cycle and signaling networks associated with inflammation, further investigation of their direct targets could provide new insights into the mechanism of their involvement in inflammatory diseases. The investigation of these miRNAs reinforces the concept that endometriosis is not only a disease of hormonal origin but is also controlled by neuroimmune mediators. The involvement of neuropeptides and chemokines suggests an interaction between pain and immune pathways in endometriosis patients, mediated by the action of sex hormones and local cytokine environments, which suggests two therapeutic strategies: (1) help reduce neuropathic pain in patients through *CGRP* receptor antagonists or monoclonal antibodies [29] and (2) control *ENA-78* by altering the expression of inhibitory miRNAs to *CGRP* expression. The reason why the expression of miRNAs and target genes differs in tissue and serum is that miRNAs in serum mainly arise from cellular secretions, apoptosis, or necrosis of various tissues, including endometriotic lesions, and may reflect systemic inflammatory processes, whereas miRNAs measured in EC and EU tissues directly reflect the local environment of inflammation and neuropathy associated with endometriotic lesions [16]. Furthermore, changes in miRNA expression in tissues may be more prominent because they are directly related to the pathophysiology of endometriotic lesions. In contrast, serum miRNA levels may be influenced by factors such as metabolic conditions or comorbidities, which explain their dilution or reduction in concentration [30].

In endometriosis, epigenetic abnormalities lead to changes in transcriptional factors, and it may be the combination of hypoxia (HIF‐1*α*), chronic inflammation (NF‐*κ*B/STAT3 activation), and elevated estrogen that blocks the transcription of miRNAs by inducing epigenetic changes (DNA hypermethylation and histone modifications) and disrupting transcription factors. This suppression of miRNAs leads to uncontrolled expression of inflammatory peptides that mutually exacerbate inflammation and hypoxia, creating a virulent pathogenic cycle [31].

cGRP is a potent vasodilator and neuroinflammatory mediator. Systemic elevation of cGRP is associated with conditions such as migraine, where cGRP causes vasodilation and trigeminal sensitization, exacerbating pain [32], and cardiovascular effects, where chronically elevated cGRP may cause hypotension or exacerbate inflammation in atherosclerosis [33]; cGRP potentiates fibrotic responses in the liver and lung through activation of TGF‐*β* [34]. While we have focused on local effects in the endometrium, we acknowledge that systemic elevation of cGRP could theoretically affect these pathways. ENA‐78 (CXCL5) is a chemokine that attracts neutrophils and promotes angiogenesis. Its systemic effects include acute lung injury, where CXCL5 increases neutrophil infiltration and worsens tissue damage [35]; rheumatoid arthritis, where increased CXCL5 in synovial fluid exacerbates joint inflammation [36]; and cancer metastasis, where CXCL5 enhances angiogenesis in tumors [37].

Although decreased expression of the studied miRNAs was observed in both tissue and serum samples, the quantitative differences between them may indicate the importance of tissue samples for investigating local molecular mechanisms and serum samples for less invasive diagnostic purposes. In future studies, it is suggested to investigate the correlation between tissue and serum miRNA levels, as well as their precise role in the transmission of pain and systemic inflammation signals. This could help determine the true potential of these miRNAs as biomarkers or therapeutic targets.

Finally, it is suggested that, considering the clear interaction between sex steroids and neuroinflammatory mediators in endometriosis patients, the hormonal background of such interventions can be investigated.

## 5. Conclusions

This study first demonstrated that the identification of microRNA expression associated with the *ENA-78* and *CGRP* genes may provide new markers for the assessment of endometriosis. *hsa-miR-5584-5p*, *hsa-miR-410-5p*, *hsa-miR-4748*, and *hsa-miR-92a-3p* modulate neuroimmune responses in endometriosis and may serve as future diagnostic or therapeutic targets. This study is a study of the interaction between inflammatory and neural mediators in endometriosis and may provide researchers with new insights into the pathophysiology of endometriosis and its treatment.

## Ethics Statement

The study was approved by the Ethics Committee of the Iran University of Medical Sciences (IR.IUMS.REC.1402.994).

## Consent

The authors have nothing to report.

## Conflicts of Interest

The authors declare no conflicts of interest.

## Author Contributions

Azam Govahi and Samaneh Rokhgireh contributed to the writing of the original draft and to the conceptualization of the work. Fateme Arjmand, Mahmood Barati, and Shahla Chaichian performed the experimental technique. Marziyeh Ajdary contributed to the conceptualization, writing the review, and editing of the paper.

## Funding

This study was funded by the Iran University of Medical Sciences, 10.13039/100012021.

## Data Availability

The underlying data supporting the results of our study can be found in the manuscript.
